# Generation and Characterization of Human Heme Oxygenase-1 Transgenic Pigs

**DOI:** 10.1371/journal.pone.0046646

**Published:** 2012-10-05

**Authors:** Hye-Jung Yeom, Ok Jae Koo, Jaeseok Yang, Bumrae Cho, Jong-Ik Hwang, Sol Ji Park, Sunghoon Hurh, Hwajung Kim, Eun Mi Lee, Han Ro, Jung Taek Kang, Su Jin Kim, Jae-Kyung Won, Philip J. O'Connell, Hyunil Kim, Charles D. Surh, Byeong-Chun Lee, Curie Ahn

**Affiliations:** 1 Transplantation Research Institute, College of Medicine, Seoul National University, Seoul, Korea; 2 Department of Theriogenology and Biotechnology, College of Veterinary Medicine, Seoul National University, Seoul, Korea; 3 Designed Animal Resource Center and Biotransplant Research Institute, Seoul National University Green-Bio Research Complex, Gangwon-do, Korea; 4 Transplantation Center, Seoul National University Hospital, Seoul, Korea; 5 Graduate School of Medicine, Laboratory of G Protein Coupled Receptors, Korea University, Seoul, Korea; 6 Molecular Pathology Center, Seoul National University Cancer Hospital, Seoul, Korea; 7 The Center for Transplant Renal Research, Westmead Millennium Institute, University of Sydney at Westmead Hospital, Westmead, New South Wales, Australia; 8 Optifarm Solution Inc., Seonggeo-eup, Cheonan, Korea; 9 The Scripps Research Institute, La Jolla, California, United States of America; 10 Division of Nephrology, Seoul National University College of Medicine, Seoul, Korea; Universidade de Sao Paulo, Brazil

## Abstract

Xenotransplantation using transgenic pigs as an organ source is a promising strategy to overcome shortage of human organ for transplantation. Various genetic modifications have been tried to ameliorate xenograft rejection. In the present study we assessed effect of transgenic expression of human heme oxygenase-1 (hHO-1), an inducible protein capable of cytoprotection by scavenging reactive oxygen species and preventing apoptosis caused by cellular stress during inflammatory processes, in neonatal porcine islet-like cluster cells (NPCCs). Transduction of NPCCs with adenovirus containing hHO-1 gene significantly reduced apoptosis compared with the GFP-expressing adenovirus control after treatment with either hydrogen peroxide or hTNF-α and cycloheximide. These protective effects were diminished by co-treatment of hHO-1 antagonist, Zinc protoporphyrin IX. We also generated transgenic pigs expressing hHO-1 and analyzed expression and function of the transgene. Human HO-1 was expressed in most tissues, including the heart, kidney, lung, pancreas, spleen and skin, however, expression levels and patterns of the hHO-1 gene are not consistent in each organ. We isolate fibroblast from transgenic pigs to analyze protective effect of the hHO-1. As expected, fibroblasts derived from the hHO-1 transgenic pigs were significantly resistant to both hydrogen peroxide damage and hTNF-α and cycloheximide-mediated apoptosis when compared with wild-type fibroblasts. Furthermore, induction of RANTES in response to hTNF-α or LPS was significantly decreased in fibroblasts obtained from the hHO-1 transgenic pigs. These findings suggest that transgenic expression of hHO-1 can protect xenografts when exposed to oxidative stresses, especially from ischemia/reperfusion injury, and/or acute rejection mediated by cytokines. Accordingly, hHO-1 could be an important candidate molecule in a multi-transgenic pig strategy for xenotransplantation.

## Introduction

The use of genetically modified pigs as a source of organs is a promising strategy to overcome severe shortages of human organs for transplantation. It is noteworthy that hyperacute xenograft rejection has been circumvented with the generation of pigs deficient in the expression of α1,3-galactosyltransferase while over-expressing human complement regulatory proteins [1], [2]. The next obstacle to overcome is acute vascular rejection (AVR), which occurs due to endothelial cell activation, intravascular coagulation, and innate immune cell-mediated inflammation. Another deleterious factor is ischemia/reperfusion injury (IRI). IRI is caused by cytotoxic mediators, such as reactive oxygen species (ROS), released during organ procurement, which also induce the expression of chemokines and adhesion molecules and the infiltration of innate inflammatory cells [3], [4].

The benefits of heme oxygenase 1 (HO-1) in transplantation are mediated by its strong anti-oxidative, anti-apoptotic and anti-inflammatory effects [5]. Other helpful functions of HO-1 are its ability to modulate the immune response, maintain microcirculation, facilitate angiogenesis, and inhibit platelet aggregation [6], [7]. As a result, many instances of HO-1 ameliorating pathological processes in transplantation have been reported.

Since Bach and his colleague firstly shown involvement of HO-1 in cardiac xenotraft model survival, several [8], studies were performed to assess the role of HO-1 in xenotransplantation. There are at least three types of benefits that HO-1 can confer. First, HO-1 provides strong protective effects for grafts against IRI [9], which can be more severe in xenografts than that seen in allografts. For instance, we previously observed that hydrogen peroxide (H_2_O_2_), a ROS, causes a much stronger up-regulation of VCAM-1 and other adhesion molecules on porcine endothelial cells (PECs) than in HUVECs [10], [11]. Much higher expression of inducible nitric oxide synthase (iNOS) and the chemokines RANTES, MIP-1a and IP-10 in concordant xeno-skin grafts than in allografts was also observed [12], [13]. The elevation of these immune mediators in the xenografts could be reduced by the over-expression of HO-1 [14]. Second, HO-1 can play an important role in reducing AVR through its anti-inflammatory function [15]. Over-expression of HO-1 significantly reduces inflammation, thrombus formation, and IgM deposition on the xenograft [16], [17]. The production of pro-inflammatory molecules such as CCR5 and ICAM-1, and NK cell activities are also reduced by HO-1. Lastly, HO-1 promotes the accommodation of xenografts by reducing xenoantibody-mediated PECs damage, as HO-1 ameliorates the human antibody response to PECs in α1,3-Gal-silenced tissues [18]. In animal experiments, the expression of HO-1 on endothelial cells and cardiac myocytes has been associated with the accommodation and prolonged xenograft survival of rodent cardiac and lung xenografts [15], [19].

In allotransplantation settings, the over-expression of HO-1 by gene therapy using an adenoviral vector has been effective in preventing IRI and the rejection of livers, kidneys and hearts [20], [21]. In xenotransplantation settings, protective effects of HO-1 have been noted in cell-based *in vitro* experiments, small animal experiments using HO-1 induced by chemicals or peptides [22], and experiments with human HO-1 transgenic (hHO-1-Tg) pigs generated by two groups [23], [24]. Based on these results, we generated a line of transgenic pigs expressing hHO-1 for xenotransplantation.

## Materials and Methods

### Animals

Isolation of neonatal porcine islet-like cluster cells (NPCCs) was from 1-3-day-old cross-breed neonatal pigs (Landrace, Yorkshire, and Duroc). White Yucatan breed miniature pigs were used for generating transgenic pigs. All animals were purchased from and maintained by Optifarm Solution (Cheonan, Korea). The protocol of animal use in this study was approved by the Institutional Animal Care and Use Committee of Seoul National University (12-2009-008-5) in accordance with the Guide for the Care and Use of Laboratory Animals of Seoul National University.

### Plasmid vector construct for hHO-1

The hHO-1 gene was amplified from cDNA of human peripheral blood mononuclear cells using Pyrobest DNA polymerase (Takara, Shiga, Japan) and a hHO-1 specific primer set (Table 1). The amplified hHO-1 gene was inserted into a pcDNA3 vector containing cytomegalovirus (CMV) promoter (Invitrogen, CA, USA) by *EcoR*I and *Xho*I digestion. Additionally, the hemagglutinin (HA) epitope was tagged using *Kpn*I and *EcoR*I to improve detection of the transgene. This study was approved by the Institutional Review Board of Seoul National University Hospital (H-0802-059-235). Informed consent was obtained from the subjects in accordance with the Declaration of Helsinki.

**Table 1:** Primer sets used for PCR and RT-PCR to detect hHO-1 in transgenic piglets.

Gene	Primer sequence (5′–3′)	Annealing temperature	PCR fragment size
HO-1^1^	F:ATGGAGCGTCCGCAACCCGACAG	62°C	867 bp
	R:TCACATGGCATAAAGCCCTACAG		
GAPDH^2^	F:CTA CTG CCA ACG TGT CGG TT	60°C	128 bp
	R:CTA CTG CCA ACG TGT CGG TT		
iNOS^3^	F:AGA GCC TCT GGA CCT CAA CA	60°C	136 bp
	R:CTC ACA GCA GAG TTC CAC CA		
RANTES^4^	F:CAT GGC AGC AGT CGT CTT TA	60°C	165 bp
	R:GGG ACA AGA GCA AGA AGC AG		
IP-10^5^	F:CAT GGA TTG CAG TCA CCA AG	60°C	73 bp
	R:AAC AGC TCG GGA TGA TGA AC		

1 *Homo sapiens* heme oxygenase 1.

2 *Glyceraldehyde-3-phosphate dehydrogenase.*

3 *Inducible nitric oxide synthase.*

4 *Regulated upon Activation, Normal T-cell Expressed, and Secreted (CCL5).*

5 *Interferon gamma-induced protein 10 (CXCL10).*

### Generation of adenoviruses containing hHO-1 and GFP

All adenoviral systems that we used have been described previously [25]. In brief, the gene encoding HA-tagged hHO-1 was cut out from pcDNA3/HA-hHO-1 by the *Kpn*I and *Xba*I restriction enzymes and inserted into pAdTrack-CMV, a shuttle vector. For homologous recombination, 0.5 µg of pAdTrack-CMV linearized with *Pme*I was mixed with 0.1 µg of pAdEasy-1, an adenoviral plasmid. The viral plasmids were digested with *Pac*I and transfected into HEK293 cells. Transfected cells were collected 9 days after transfection, and viral lysates were obtained by three cycles of freezing and thawing (Ad-hHO-1). Adenoviruses containing the GFP gene were also produced as a control (Ad-GFP).

### Isolation and culture of NPCCs

The pancreas was eviscerated from anesthetized neonatal pigs and placed into Hanks' balanced salt solution (Invitrogen, CA, USA). The pancreas was sliced, digested with 2.5 mg/ml collagenase V (Sigma, MO, USA), and filtered through a 500 µm stainless-steel mesh. The filtrate containing NPCCs was cultured for 7–10 days at 37°C, 5% CO_2_ in Ham's F10 medium (Gibco®, NY, USA) supplemented with 180 mg/dL glucose, 50 µM isobutylmethylxanthine, 0.5% bovine serum albumin (Fraction V), 2 mM L-glutamine, 3 mM CaCl_2_, 10 mM nicotinamide (all from Sigma, MO, USA), and 1% penicillin-streptomycin solution (Invitrogen, CA, USA). For each experiment, pancreas from 1 to 3 piglets were pooled and used for analysis. Each experiment was repeated 3 times.

### Preparation of porcine fibroblasts

To produce the hHO-1-Tg pigs, fibroblasts were isolated from the skin of porcine fetuses at day 35 of gestation were used. For analyzing effects of hHO-1 in hHO-1-Tg pig, fibroblasts obtained from ear tissue of F0 generation of hHO-1-Tg pig were used. Since our pig did not express hHO-1 in endothelial cells (data not shown), skin fibroblasts were used for *in vitro* function experiments of the hHO-1 transgene. The tissues were washed and minced in Ca^2+^- and Mg^2+^-free phosphate-buffered saline (PBS; Invitrogen, CA, USA) and then dissociated by 0.25% Trypsin/EDTA solution (Invitrogen, CA, USA) for 30 min at 37°C. Trypsinized cells were washed and cultured in Dulbecco's modified Eagle's medium (DMEM; Invitrogen, CA, USA) supplemented with 10% fetal bovine serum (Invitrogen, CA, USA) and 1% Antibiotic-Antimycotic Solution (Invitrogen, CA, USA) at 39°C in a humidified atmosphere of 5% CO_2_ and 95% air.

### Oxidative or inflammatory stimulation of NPCCs and hHO-1-Tg fibroblasts

NPCCs were plated at a density of 500 islet equivalents (IEQ; one IEQ = an average diameter of 150 µm) per well in a six-well dish and infected with adenovirus at the multiplicities of infection indicated. Various concentrations (0, 50, 100, 200, or 400 µM) of H_2_O_2_ for oxidative stimulation or 25 ng/ml of recombinant human tumor necrosis factor α (hTNF-α eBioscience, CA, USA) and 10 µg/ml cycloheximide (CHX; Sigma, MO, USA) were applied for 24 hr. Zinc protopotphorine IX (Zn(II)PPIX, Sigma, MO, USA), an inhibitor of HO-1, was also treated as experimental design at the concentration of 20 µM. To investigate the benefits of the hHO-1-Tg pig, fibroblasts from the F0 generation of transgenic pig were plated at a density of 1×10^5^ cells/well in a 12-well plate and treated with various concentrations (0, 200, 400, or 800 µM) of H_2_O_2_ or with 20 ng/ml of hTNF-α and 10 µg/ml CHX for 1 or 15 hr, respectively. Effective concentration of hTNF-α and CHX for NPCCs was chosen by dose titration for fibroblasts experiment was based on a previous study [26]. In the fibroblasts sets, we conducted experiments for dose titration (hTNF-α 10 ng/ml and CHX 10 µg/ml, hTNF-α 20 ng/ml and CHX 10 µg/ml, hTNF-α 50 ng/ml and CHX 10 µg/ml; 4, 6, 12, 13, 14, 15, and 18 hr, data not shown) and the optimum concentration at hTNF-α 20 ng/ml and CHX 10 µg/ml was chosen for further analysis. Concentration of Zn(II)PPIX was also determined by dose titration (0, 20, 40, 60 µM for 24 hr; data not shown). Each experiment was repeated 3 times.

### Cell viability analysis

Cell viability was determined with a Cell Counting Kit-8 (CCK-8; Dojindo Laboratories, Kumamoto, Japan) according to manufacturer's instructions. Briefly, CCK-8 solution of one tenth of the volume of the medium was added to each well of the plate. The NPCCs were incubated for 4 hr after the addition of the CCK-8 solution. Fibroblasts were incubated for 1 hr after the addition of the CCK-8 solution. The absorbance was measured at 450 nm using a microplate reader.

### Assays for apoptosis detection

The resistance of hHO-1 against apoptotic damage after challenging with H_2_O_2_ or hTNF-α and CHX was determined by a Caspase-Glo® 3/7 assay (Promega, WI, USA) following the manufacturer's instructions. Trypsinized fibroblasts or 500 IEQ (1×10^5^ cells) of NPCCs, were washed twice with PBS and then resuspended in 100 µl PBS. The samples were mixed with 100 µl of the Caspase-Glo® 3/7 reagent, and after incubation for 10 min at room temperature, the level of caspase was measured by luminometer.

Apoptosis of NPCCs or fibroblasts was also analyzed using an Annexin V Apoptosis Detection Kit (BD Biosciences, CA, USA) with 7-AAD or propidium iodide (BD Biosciences, CA, USA) following the manufacturer's instructions. Briefly, 5 µl of PE–annexin V conjugate and 5 µl of 7-AAD (BD Bioscience, CA, USA) or 5 µl of propidium iodide were added to each sample tube and incubated for 15 min at room temperature in the dark. After that, 400 µl of binding buffer was added to each sample tube and the cells were analyzed by flow cytometry (FACS Calibur; BD Biosciences, CA, USA).

### Production of hHO-1-Tg pigs

Transgenic pigs were produced by SCNT as described in previous studies with slight modifications [27], [28]. Briefly, the pcDNA3/HA-hHO-1 plasmid was linearized by *Sca*I and introduced into fetal fibroblasts by electroporation. After selection using 1 mM of G418 for 2 weeks, highly hHO-1 expressing cells were electrically fused with *in vitro* matured, enucleated porcine oocytes. After 30 minutes, the reconstructed embryos were artificially activated by electric pulses and then cultured *in vitro* for 1–2 days. Ninety to 120 embryos were transferred to oviduct an estrus-synchronized surrogate pig (Landrace x Yorkshire) by laparotomy. Pregnancy was monitored by ultrasonography, and the transgenic pigs were delivered by caesarian section around day 114 of gestation.

### Identification of transgenic hHO-1

The insertion and expression of the hHO-1 transgene were analyzed by genomic DNA PCR, RT-PCR and western blot. Genomic DNA and total RNA were isolated using commercial kits (iNtRON Biotechnology, Seongnam, Korea). For western blot analysis, the samples were transferred to polyvinylidene fluoride membranes by electroblotting using the wet transfer system. After blocking in TBST buffer (10 mM Tris-HCl, pH 7.5, 150 mM NaCl, and 0.05% Tween 20) containing 5% (w/v) skim milk, the membranes were incubated with hHO-1 antibody (1∶200; Abcam, MA, USA) and/or HA antibody (1∶4,000; Abcam, MA, USA), which was followed by another incubation with anti-rabbit or anti-mouse immunoglobulin G coupled with horseradish peroxidase, as required.

### Immunohistochemistry

Tissues harvested from various organs were fixed with 4% paraformaldehyde and embedded in paraffin. Sections (4 µm) were cut and air-dried on gelatin-coated slides and reacted with hHO-1 (1∶100; Abcam, MA, USA) or HA antibodies (1∶1,000; Abcam, MA, USA). For staining, avidin-biotin-complex/diaminobenzidine immunohistochemistry method was followed (ABC kit; Dako, Glostrup, Denmark).

### Measurement of Reactive Oxygen Species

The levels of ROS were examined using dichlorodihydrofluorescein diacetate (DCHF-DA; Sigma, MO, USA). After treatment with H_2_O_2_, the fibroblasts were cultured with 25 µM DCHF-DA for 15 min. After incubation, single cells were harvested using TrypLE Express (Invitrogen, CA, USA). The level of ROS was analyzed by flow cytometry at 485 nm (FACS Calibur; BD Biosciences, CA, USA).

### Real-time RT PCR

Real-time quantitative PCR was performed in triplicate in 384-well plates; each 20 µl reaction consisted of 2xSYBR Green PCR Master Mix (PE Applied Biosystems, CA, USA), and 10 pmol/µl forward and reverse primers corresponding to iNOS, RANTES, IP-10, or GAPDH (Table 1). The thermal cycling conditions were 50°C for 2 min and 95°C for 10 min followed by 40 cycles of 95°C for 30 s, 60°C for 30 s, and 72°C for 30 s. The real-time PCR analysis was performed on an Applied Biosystems Prism 7900 Sequence Detection System (PE Applied Biosystems, CA, USA).

### Statistical Analysis

Student's t-test was used for statistical analyses between each group. Data were presented as mean ± SEM and the p-value less than 0.05 were considered significant. All the analysis was performed using Prism software (Version 4.03; GraphPad, CA, USA).

## Results

### Protective effect of hHO-1 against oxidative stress and inflammatory cytokine-mediated apoptosis in NPCCs

**Figure 1:**
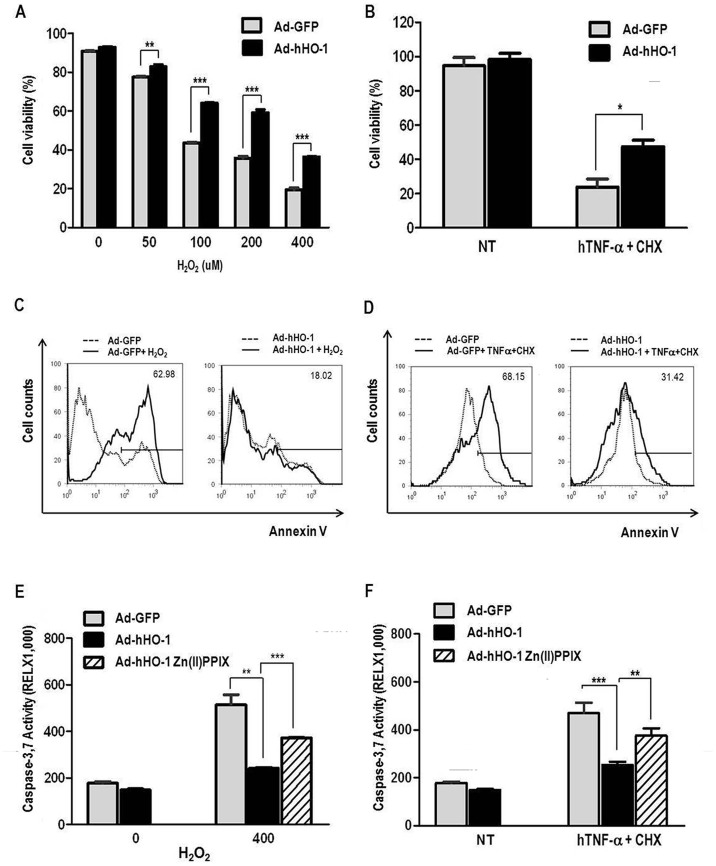
Impact of Ad-hHO-1 on H_2_O_2_ or hTNF-α and CHX-induced cell death and apoptosis in NPCCs. **A:** Ad-hHO-1 increased cell viability in H_2_O_2_-treated NPCCs. NPCCs were transduced with Ad-hHO-1 for 24 hr before the addition of H_2_O_2_ (0, 50, 100, 200, or 400 µM). After incubating the cells with H_2_O_2_ for an additional 24 hr, cell viability was measured by CCK-8. **B:** After treatment with hTNF-α and CHX cell viability was measured by CCK-8. An increased proportion of viable cells were detected in the Ad-hHO-1 group compared with the Ad-GFP control group. NPCCs were infected with Ad-hHO-1 for 24 hr before the addition of hTNF-α (25 ng/ml) and CHX (10 µg/ml). After treatment with hTNF-α and CHX, the NPCCs were incubated for an additional 24 hr. **C:** FACS analysis of apoptosis in NPCCs after treatment with 400 µM H_2_O_2_. A lower proportion of apoptotic cells were noted in Ad-hHO-1 (50 MOI) infected NPCCs relative to the Ad-GFP control. The NPCCs were treated with H_2_O_2_ (400 µM) and FACS analysis was performed using annexin V-PE. Each experiment was repeated three times (data not shown). **D:** FACS analysis of apoptosis in NPCCs after treatment with hTNF-α and CHX for 24 hr. A smaller proportion of apoptotic cells was detected in Ad-hHO-1 (200 MOI)-infected NPCCs than in the Ad-GFP (200 MOI) group (p*<0.05)*. After treatment with hTNF-α and CHX, apoptosis of cultured NPCCs was measured by FACS using annexin V. The numbers indicate the proportion of apoptotic cells in the NPCCs after 24 hr of incubation with hTNF-α and CHX. **E:** Lower activity of caspase 3/7 in Ad-hHO-1-infected NPCCs relative to Ad-GFP-infected NPCCs after 24 hr of incubation with H_2_O_2_ (400 µM). After Zn(II)PPIX treatment, Ad-hHO-1 increased activity of caspase 3/7. Each experiment was repeated three times. (* p<0.05, ** p<0.01, *** p<0.001, NT: no treatment). **F:** The activities of caspase 3/7 were measured in Ad-hHO-1-infected NPCCs. The protective effect of Ad-hHO-1 in the NPCCs compared with Ad-GFP after 24 hr of incubation with hTNF-α and CHX. Each experiment was repeated three times. (** p<0.01, *** p<0.001).

To investigate the protective effects of hHO-1 over-expression, we transduced hHO-1 or GFP into NPCCs using an adenoviral vector (Ad-hHO-1 or Ad-GFP, respectively). The western blot analysis demonstrated that hHO-1 was successfully introduced into NPCCs. These NPCCs were tested for susceptibility to H_2_O_2_-induced apoptosis during a 24 hr period. NPCCs transduced with hHO-1 survived significantly better than control GFP-transduced NPCCs, especially at higher concentrations of H_2_O_2_ (Fig. 1A). NPCCs transduced with hHO-1 were tested for susceptibility to hTNF-α-induced apoptosis in the presence of CHX for 24 hr. As observed above for H_2_O_2_, hHO-1-transduced NPCCs survived significantly better than control GFP-transduced cells (Fig. 1B). Thus, hHO-1-transduced NPCCs had a considerably lower number of annexin V-positive cells (Fig. 1C; 16.23±2.79% *vs*. 42.33±11.48%, p<0.05, Fig. 1D; 26.17±6.13% *vs*. 40.38±8.26%, p<0.01) and lower levels of caspase 3/7 activity than GFP-transduced NPCCs (Fig. 1E; 240.98±3.27% *vs*. 514.851±42.21%, p<0.01, Fig. 1F; 252.45±14.11% *vs*. 468.78±43.91%, p<0.001). After Zn(II)PPIX treatment, Ad-HO-1 showed increased levels of caspase 3/7 compared with untreated Ad-HO-1 (Fig. 1E; 372.23±1.88%, Fig. 1F;375.11±31.17%).

### Generation and validation of hHO-1-Tg pigs

**Figure 2:**
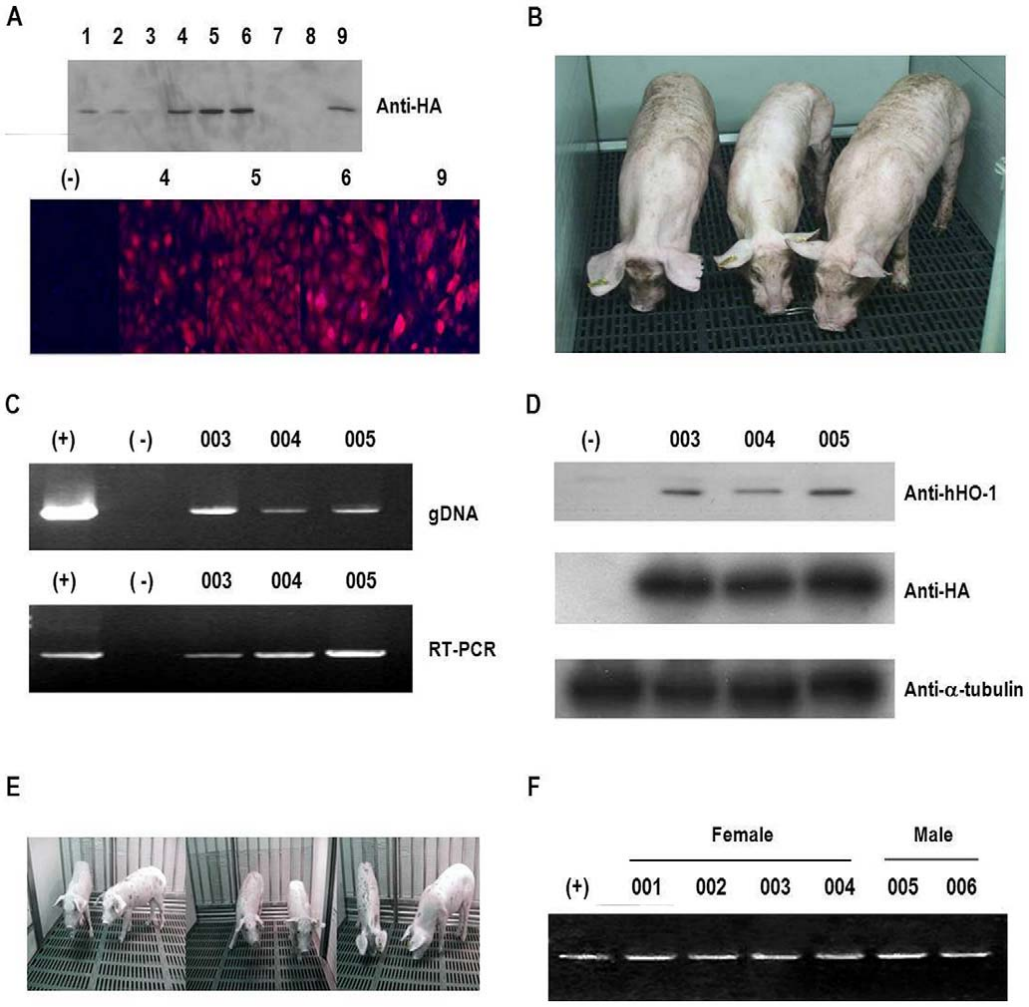
Generation of hHO-1 transfected donor cell lines and hHO-1-Tg pigs. **A:** Establishment of hHO-1-expressing cells using O-type white Yucatan pig fibroblasts. Expression of hHO-1 was identified by western blot analysis and immunocytochemistry. A donor cell colony with a high level of expression was chosen, and nuclear transfer was performed. **B:** Three hHO-1-Tg pigs were generated (F0; #003, #004, and #005). **C:** Expression of the hHO-1 gene was confirmed by genomic DNA PCR and RT-PCR. Genomic DNA recovered from blood was analyzed by PCR (top lane). RT-PCR analysis of whole blood isolated from the HA-hHO-1-Tg pigs is shown in the bottom lane (#003, #004, and #005). Positive control: hHO-1-transfected cells (MPN3); negative control: normal pig. **D:** Western blotting was performed in ear tissue from the transgenic pig using anti-hHO-1 and anti-HA antibodies. The α-tubulin was used as a control. **E**: Six hHO-1-Tg progenies (F1; 4 females and 2 males) pigs were generated after bred of one F0 pig with wild type female. **F**: Expression of the hHO-1 gene was confirmed by RT-PCR analysis of whole blood isolated from the HA-hHO-1-Tg pigs. Positive control (+): hHO-1-transfected cells (MPN3). Negative control (−): wild type pigs. All the 6 piglets were confirmed as transgenic.

After introducing the hHO-1 transgene into porcine fetal fibroblasts, the expression of the transfected gene was confirmed by western blot and immunocytochemistry (Fig. 2A). Only highly expressing cell colonies were expanded and used for SCNT. In total, 617 reconstructed embryos were transferred to 6 surrogate pigs. Among them, three surrogates were detected to be pregnant and 3 cloned pigs were produced from two of them (Table. 2). All 3 hHO-1-Tg pigs were healthy and grew normally in a SPF facility (Fig. 2B). The presence and expression of the hHO-1 transgene in the cloned pigs were detected by genomic DNA PCR, RT-PCR, and western blot analysis (Fig. 2C and D). Additionally, expression of the hHO-1 transgene from fibroblasts was assessed by flow cytometry and 69.3±3.07% of the cells is detected as hHO-1 positive (data not shown). After puberty, one of the transgenic pigs was bred with a wild-type female pig, and 6 progeny (4 females and 2 males) were born. All piglets were confirmed as transgenic by PCR analysis (Fig. 2E and F).

**Table 2:** Production of cloned hHO-1 transgenic pigs.

ID	No. of transferred embryos	Pregnancy	Born piglets	Current Status
1	90	No	-	-
2	90	No	-	-
3	103	Yes	1	Alive
4	113	Yes	2*	Alive
5	120	No	-	-
6	101	Yes	0	Abortion

* One of them was sacrificed at 456 days to analyze hHO-1 protein expression pattern in various organs.

### Expression pattern of hHO-1 in various organs of the F0 and F1 transgenic pig by western blot

**Figure 3:**
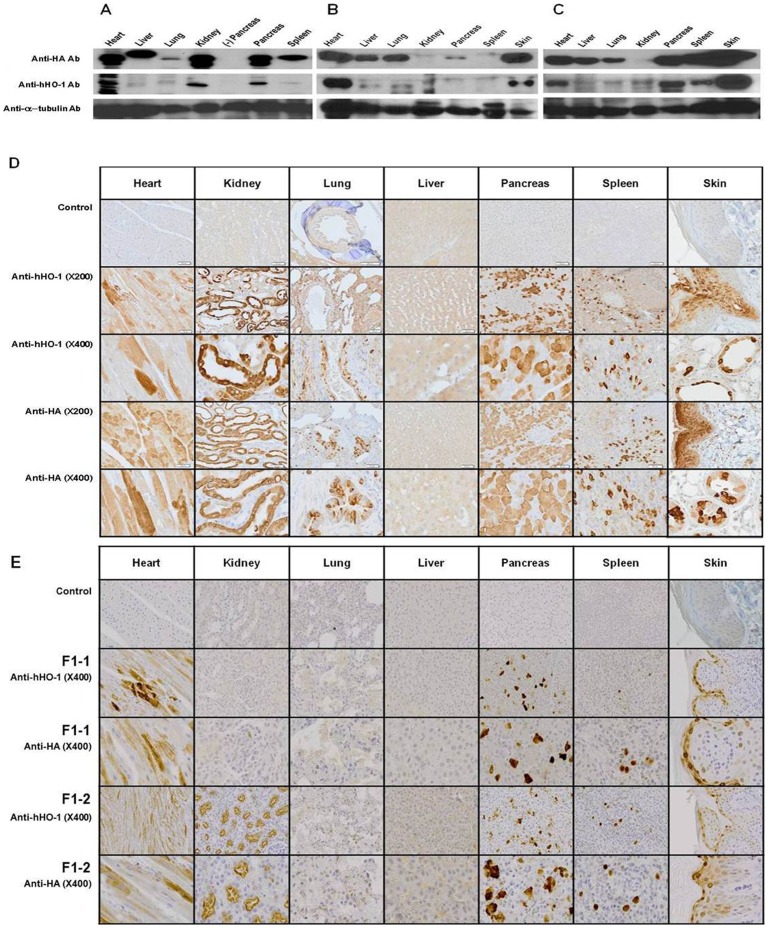
Tissue distribution of hHO-1 protein in hHO-1-Tg pig tissues. **A:** The tissue distribution of the hHO-1 protein was analyzed in various organs by western blot analysis in a 465-day-old male hHO-1 F0 pig. Western blot was performed on heart, kidney, lung, liver, pancreas, spleen, and skin tissue. **B:** The tissue distribution of the hHO-1 protein was analyzed in various organs by western blot analysis in a 74-day-old male hHO-1 F1 pig. Western blot was performed on heart, kidney, lung, liver, pancreas, spleen, and skin tissue. **C:** The tissue distribution of the hHO-1 protein was analyzed in various organs by western blot analysis in a 74-day-old male hHO-1 F1 pig. Western blot was performed on heart, kidney, lung, liver, pancreas, spleen, and skin tissue using anti-hHO-1 (1∶1,000) and anti-HA antibodies (1∶4,000). Twenty micrograms of protein were loaded into each lane as indicated by the α-tubulin band. **D:** Immunohistochemistry of heart, kidney, lung, liver, pancreas, spleen, and skin tissues of hHO-1 F0 pig were performed to analyze expression patterns of hHO-1 and HA. Paraffin sections of various organs were stained with anti-HA antibody (1∶1,000), anti-hHO-1antibody (multiple organs: 1∶100) and control (anti-hHO-1 antibody, anti-HA antibody: data not shown). **E:** Immunohistochemistry of heart, kidney, lung, liver, pancreas, spleen, and skin tissues of hHO-1 F1 (n = 2) were performed to analyze expression patterns of hHO-1 and HA. Paraffin sections of various organs were stained with anti-HA antibody (1∶1,000), anti-hHO-1antibody (multiple organs: 1∶100) and control (anti-hHO-1 antibody, anti-HA antibody: data not shown).

At 465 days old one of the male transgenic pigs (F0) was sacrificed to analyze the expression pattern of hHO-1 in various organs by western blot and immunohistochemistry. The expression level of the hHO-1 transgene was considerably variable among these organs (Fig. 3A). By western blot, the expression of hHO-1 was high in the heart, kidney, and pancreas and low in the liver, lung, and spleen. Two 74 days old male transgenic pigs (F1) were sacrificed to analyze the expression pattern of hHO-1 in the organs by western blot (Fig. 3B and C). Expression of hHO-1 and HA was similar to F1, except showing reduced expression in the kidney.

### Tissue distribution of hHO-1 in the various organs of the transgenic pig by immunohistochemistry

The presence of hHO-1–positive cells was confirmed in F0 and F1 tissues by immunohistochemistry (Fig. 3D and E). Neither hHO-1 nor HA were detected in any tissue from the age- and sex-matched wild-type pig (data not shown), however, both molecules were detectible in the transgenic pigs. Interestingly, the expression levels were not uniform even in the same organ. In the kidney, anti-HA and anti-HO-1 staining showed positive staining in the brush border of proximal tubules and strong positivity in the cytoplasm of distal tubules and collecting ducts, however, negative in glomeruli. In the heart, anti-HA and anti-HO-1 staining reveals a heterogeneous positivity in cardiomyocytes in contrast to absence of staining in control cases. In the pancreas, anti-HA and anti-HO-1 revealed positive staining in pancreatic acini in a mosaic pattern but not in the islet and ductal cells. Endothelial cells in each organ stained negative.

### Antioxidant effect of hHO-1 in fibroblasts derived from the transgenic pig

**Figure 4:**
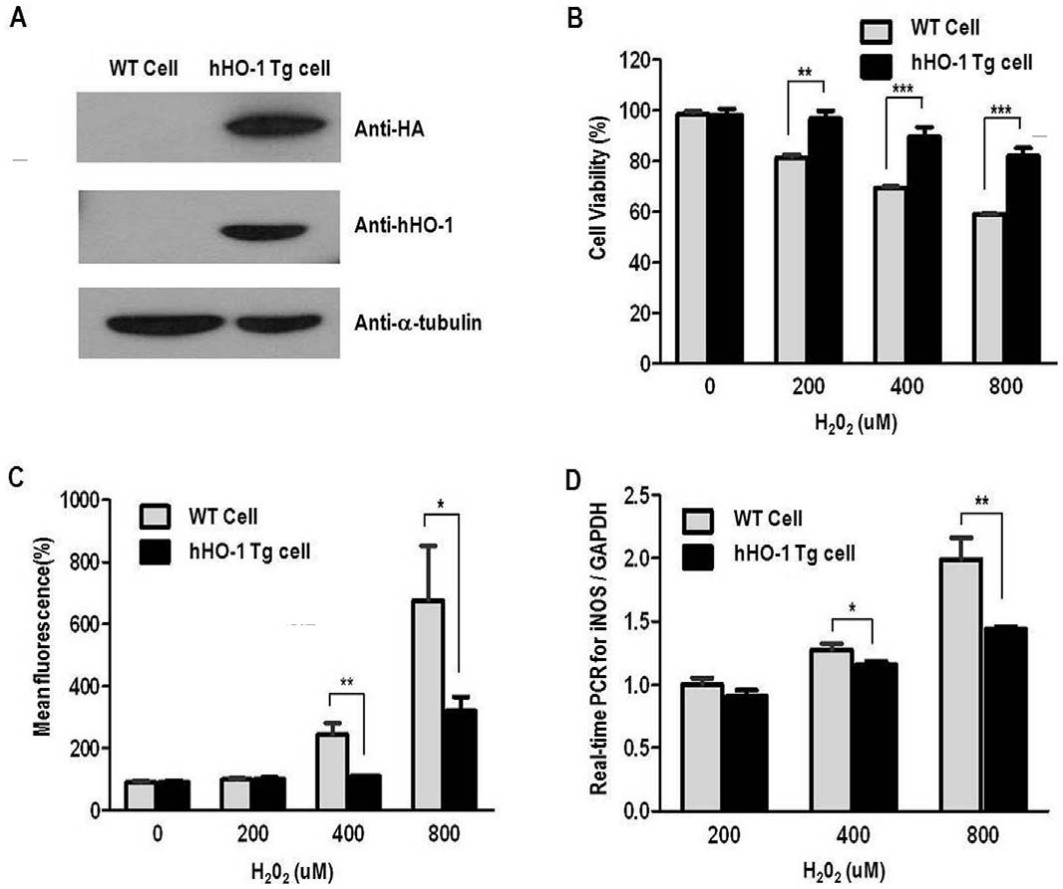
Anti-oxidant effects of fibroblasts from the hHO-1-Tg pig. **A:** Western blot analysis for hHO-1 and HA in the hHO-1-Tg fibroblasts using anti-HO-1 (1∶200) and anti-HA (1∶4,000) antibodies. The α-tubulin was used as a control. **B:** Cell viability was higher in the hHO-1-Tg fibroblasts compared to the wild-type. After H_2_O_2_ (0, 200, 400 or 800 µM) treatment, cell viability was slightly better in the hHO-1-Tg fibroblast than in the wild-type fibroblasts. Cell viability was measured by CCK-8 after induction with H_2_O_2_ for an additional 1 hr. **C:** Level of ROS was significantly reduced in the hHO-1-Tg fibroblasts in comparison to the wild type fibroblasts. After H_2_O_2_ treatment, ROS production was significantly reduced in the hHO-1-Tg fibroblasts compared with the wild-type fibroblasts. Fibroblasts obtained from ear tissues were incubated with 25 µM DCFH-DA for 15 minutes at 37°C, and ROS were measured using FACS. **D:** Expression of the iNOS gene by real-time PCR in hHO-1-Tg fibroblasts compared with the wild-type fibroblasts. The threshold cycle (Ct) value was defined as the number of PCR cycles at which the fluorescence crossed a fixed threshold above baseline. For relative quantification, the ΔΔCt method was used to measure fold changes of cDNA. (* p<0.05, ** p<0.01, *** p<0.001).

We next examined the protective effects of hHO-1 against ROS- and hTNF-α–mediated apoptosis and against inflammation using fibroblasts obtained from ear tissues of hHO-1-Tg pig. Human HO-1 was highly expressed in transgenic pig fibroblasts, whereas no expression was detected in the wild-type fibroblasts (Fig. 4A). After treatment with H_2_O_2_, the hHO-1-Tg fibroblasts showed a significantly higher viability compared with the wild-type (Fig. 4B). The levels of ROS (Fig. 4C) and iNOS (Fig. 4D) were significantly lower in the hHO-1-Tg fibroblasts than the wild-type cells.

### Anti-apoptotic effect of hHO-1 in transgenic porcine fibroblasts

**Figure 5:**
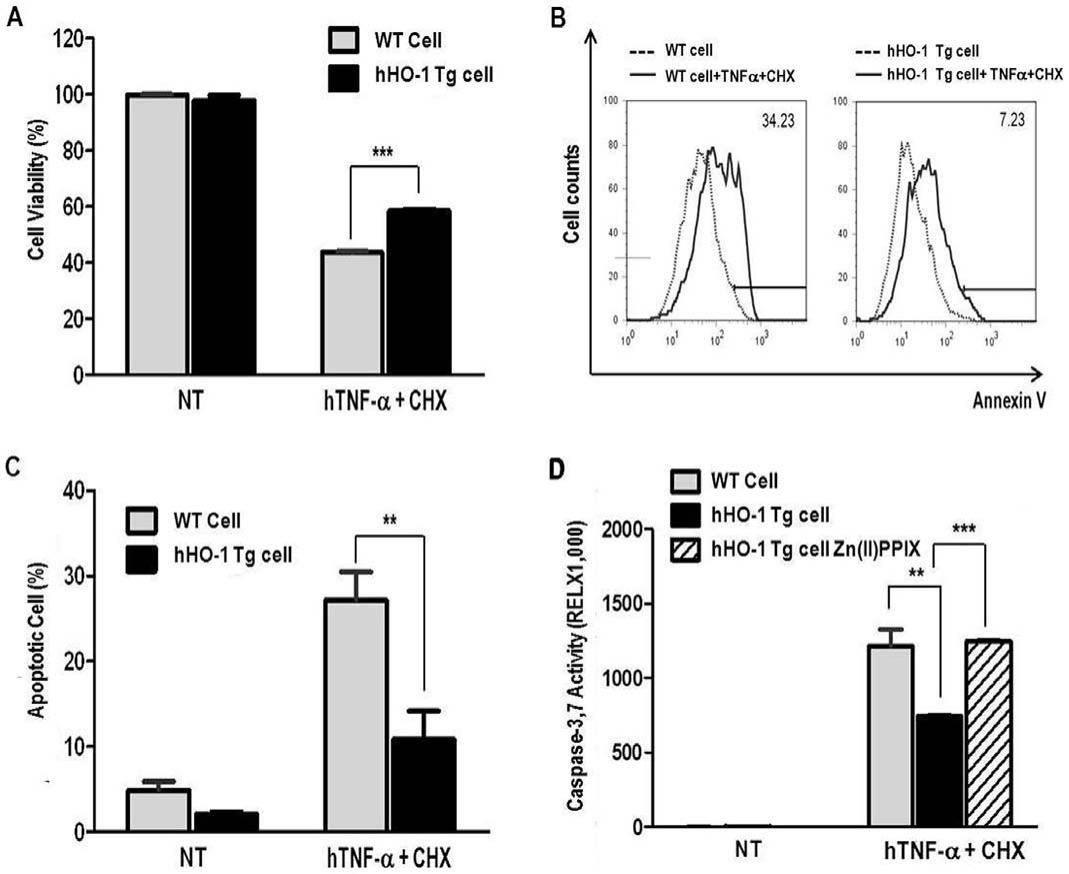
Anti-apoptotic effects of fibroblasts from the hHO-1-Tg pig. **A:** Cell viability was measured using the CCK-8. After induction with hTNF-α (25 ng/ml) and CHX (10 µg/ml) for 15 hours, the hHO-1 group had a lower percentage of viable cells. **B and C:** FACS analysis of hTNF-α and CHX-mediated apoptosis in fibroblasts. Apoptosis was analyzed using annexin V staining. The numbers indicate the proportion of apoptotic cells in the NPCCs after 15 hr incubation with hTNF-α and CHX. **D:** Activities of caspase 3/7 were measured in fibroblasts. Human HO-1 protected transgenic fibroblasts from apoptosis after 15 hr of incubation with hTNF-α and CHX. After Zn(II)PPIX treatment, Ad-hHO-1 increased activity of caspase 3/7. Each experiment was repeated three times. (* p<0.05, ** p<0.01, *** p<0.001, NT: no treatment).

After stimulation with hTNF-α and CHX for 15 hr, hHO-1-Tg fibroblasts showed significantly higher viability than wild-type fibroblasts (Fig. 5A; 58.48±0.54% *vs*. 43.67±0.37, p<0.001). Annexin V-positive cells were significantly decreased in hHO-1-Tgfibroblasts compared with the wild-type cells (Fig. 5B and C; 10.83±3.32% *vs*. 27.14±3.31%, p<0.01). Caspase 3/7 activities were also significantly lower in the hHO-1-Tg cells (Fig. 5D; 743.30±6.98% *vs*. 1211.83±114.29, p<0.01). After Zn(II)PPIX treatment, hHO-1-Tg cells showed similar levels of caspase 3/7 compared to wild-type cells (Fig. 5D; 1247.49±1.83%).

### Anti-inflammatory effect of hHO-1 in transgenic porcine fibroblasts

**Figure 6:**
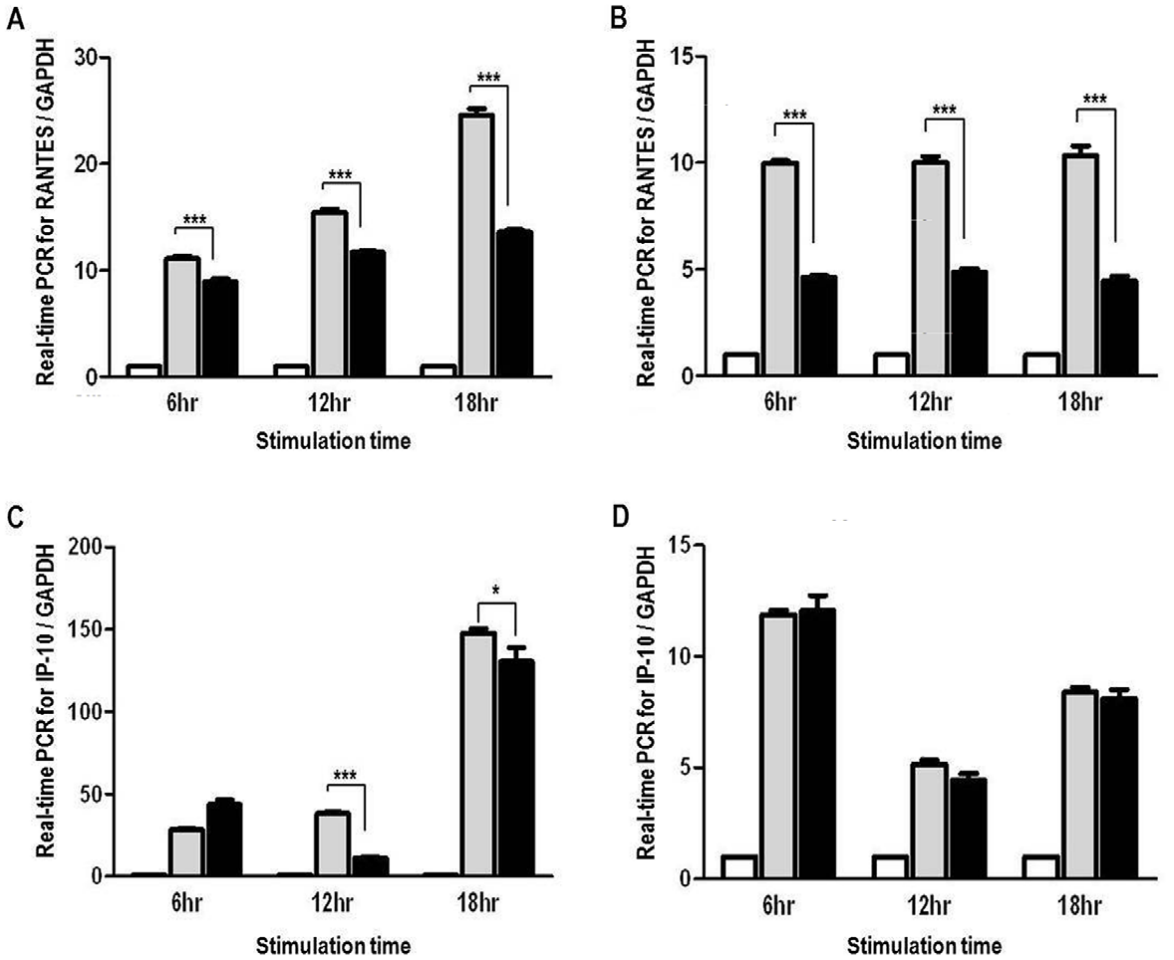
Anti-inflammatory effects of fibroblasts from the hHO-1-Tg pig. **A:** Expression of the RANTES gene in hHO-1-Tg fibroblasts compared with wild-type fibroblasts in response to hTNF-α stimulation (20 ng/ml) for 6, 12 or 18 hr. RANTES mRNA was measured by real-time PCR. **B:** Expression of the RANTES gene in hHO-1-Tg fibroblasts compared with the wild-type fibroblasts in response to LPS stimulation (1 µg/ml) for 6, 12, or 18 hr. RANTES mRNA was measured by real-time PCR. **C:** Expression of the IP-10 gene in hHO-1-Tg fibroblasts compared with the wild-type fibroblasts in response to hTNF-α stimulation (20 ng/ml) for 6, 12 or 18 hr. IP-10 mRNA was measured by real-time PCR. **D:** Expression of the IP-10 gene in hHO-1-Tg fibroblasts compared with the wild-type fibroblasts in response to LPS stimulation (1 µg/ml) for 6, 12, or 18 hr. IP-10 mRNA was measured by real-time PCR. (* p<0.05, *** p<0.001, White bars: control, gray bars: WT cell, black bars: HO-1-Tg cells).

Fibroblasts from the transgenic and wild-type pigs were incubated with hTNF-α or LPS for 6, 12, or 18 hr. After stimulation, significantly lower induction of RANTES was observed in the hHO-1-Tg fibroblasts than the wild-type fibroblasts by real-time PCR (Fig. 6A; 6 hr, 8.94±0.27 *vs*. 11.11±0.27; 12 hr, 11.71±0.12 *vs*. 15.44±0.34; 18 hr, 13.61±0.20 *vs*. 24.58±0. and B; 6 hr, 4.63±0.08 *vs*. 9.99±0.11; 12 hr, 4.89±0.16 *vs*. 10.02±0.28; 18 hr, 4.47±0.22 *vs*. 10.35±0.45; p<0.001). The induction of IP-10 after hTNF-α treatment was also lower in hHO-1-Tg fibroblasts than in wild-type fibroblasts at 12 and 18 hr (Fig. 6C; 12 hr, 11.08±0.79 *vs*. 38.25±0.67, p<0.001; 18 hr, 130.50±8.26 *vs*. 147.73±2.46, p<0.05). However, there was no significant difference between the hHO-1 group and the control group in IP-10 expression after stimulation with LPS (Fig. 6D).

## Discussion

Human HO-1 is regarded as a promising cytoprotector in transplantation because hHO-1 suppresses the generation of oxygen radicals, inhibits TNF-α-induced apoptosis [29], and has anti-inflammatory effects. The protective functions of HO-1 depend on products generated by HO-1-mediated degradation of heme. HO-1 degrades heme into biliverdin, carbon monoxide (CO), and iron. Biliverdin is reduced into bilirubin, which is a strong antioxidant, inhibits endothelial activation, and complements activation and leukocyte infiltration [30], [31]. In addition, CO has anti-apoptotic and anti-inflammatory effects through the activation of the p38 MAPK pathway [32]. CO also elevates intracellular cGMP, induces vasodilation and inhibits platelet aggregation [33]. Lastly, iron rapidly up-regulates the expression of ferritin, which chelates metal ions and protect cells from ROS-mediated injuries [9], [34].

Delivering HO-1 into the allografts is difficult in clinical settings. For example, cobalt-protoporphyrin, a strong inducer of HO-1 synthesis, is difficult to standardize for clinical use [35]. If given parenterally, most HO-1 inducers can provoke serious side effects such as porphyria [5]. Enhancing hHO-1 expression in xenografts is much easier than in allografts because the generation of hHO-1-Tg pigs is technically feasible. A hHO-1-Tg pig was first described in 2007 by Yang et al. [23]. They produced an human decay accelerating factor (hDAF)/hHO-1 double-Tg pig and reported that addition of hHO-1 gene provided little advantage in *ex-vivo* kidney perfusion model. However, PECs isolated from the hDAF/hHO-1 double-Tg pig did not display additional cytoprotective traits against monkey and human serum compared to PECs from hDAF-Tg pig [36]. In 2011, Petersen et al. also reported a hHO-1-Tg pig and they found PECs isolated from a hHO-1-Tg pig showed resistance to hTNF-α-mediated inflammatory responses and down-regulation of adhesion molecules such as E-selectin, ICAM-1, and VCAM-1 [24]. They also reported a beneficial effect of hHO-1-Tg on IRI in kidney *ex-vivo* perfusion and cardiac ischemia models [37] although the expression of hHO-1, driven by the simian vacuolating virus 40 (SV40) promoter, was weak in heart.

Because the cytoprotective effect of hHO-1 is controversial in the previous studies and not clear in porcine pancreatic cells, we first examined the impact of hHO-1 over-expression in NPCCs by Ad-hHO-1 transduction. We used NPCCs in this study because it is easy and less costly to isolate compared to the adult islets but still functional after transplantation in nonhuman primates [38]. Though, NPCCs are more resistant to ischemic injury when compared with adult porcine islets [39], Ad-HO-1 transduction effectively protected NPCCs from apoptosis induced by both oxidative (H_2_O_2_) and inflammatory stimuli (hTNF-α and CHX). Our results were similar to the previous reports performed in mice, rats and humans that the induction of HO-1 protects islets from damage [40], [41]. These results suggest that hHO-1 overexpression in porcine islets will be a promising approach to promote successful xenotransplantation as islets are extremely sensitive to hypoxic stress, and more prone to suffer ischemic damage during isolation and encapsulation process [42].

Based on the preliminary *in vitro* data, we generated three founder Tg pigs and six F1 piglets in which hHO-1 was driven by a CMV promoter. Transgenic hHO-1 was expressed well in most organs, including heart, lung, and kidney. Information about tissue localization is essential for choosing grafts or tissues. In our pig, the hHO-1 gene was expressed at high levels in cardiac muscle, kidney tubular epithelial cells, pancreatic acini, and skin, and was also expressed in interstitial tissue. These locations are essential for a xenograft to survive the injury induced by periods of ischemia, reperfusion. On the other hand, the differential expression of the transgene in subsets of cells within various organs even under the control of a global CMV promoter was noteworthy. The possible reasons for the variable expression pattern of the transgene are the regulatory elements around the transgene insertion site and the differential methylation status of the promoter region in different tissues and cell lineages. Kong et al. observed similar instable expression of transgene in their transgenic pigs expressing CMV promoter driven green fluorescent protein (GFP) [43]. In the report they find correlation of GFP expression with CMV promoter methylation. This phenomenon was also found in previous reports using different promoters such as SV40 promoter where expression was restricted to the kidney [24] and chicken beta-actin (CAG) promoter where expression was restricted to the heart and skeletal muscle [44]. High expression of hHO-1 in heart and kidney in the present study is promising. However its use in islet xenotransplantation is somewhat doubtable due to the negligible expression in the pancreatic islets. The future use of promoters of mammalian origin or the use of organ specific promoters, including the insulin promoter, will be considered to obtain better expression of hHO-1 in islets. Expressions of the transgene were also different between F0 and F1 generations of hHO-1-Tg pigs. In the F1 generation (n = 2), the expression in the kidney of hHO-1 was weak compared with that in the F0. It was demonstrated previously that high expression of the HO-1 transgene can be lethal because it may block the cell signals initiated by ROS and also may inhibit several differentiation pathways [45]. Therefore, it may be a better option to use F1 organs with low constitutive expression of hHO-1 as founder animals for xenotransplantation.

In this paper, we confirmed that hHO-1-Tg fibroblasts were resistant to ROS and cytokine mediated cell death and had anti-inflammatory effects. After H_2_O_2_ stimulation, the apoptotic pathway is activated in accompaniment with TNF-α induction, IL-1β secretion, and Toll-like receptors stimulation. When transplanted, ROS can be generated rapidly by the NAPDH oxidase complex present in neutrophils, monocytes, macrophages, and T lymphocytes [46]. As observed in our study, after treating with H_2_O_2_, hHO-1-Tg fibroblasts had reduced ROS generation accompanied by improved cell viability demonstrating its antioxidant property. We also observed that TNF-α-mediated apoptosis was reduced in hHO-1-Tg fibroblasts. HO-1 expression induced by cobalt protoporphyrin can reduce cell death by blocking caspase enzyme activity [26]; however, it was unclear whether HO-1 influences caspase activity directly because metalloporphyrins can inactivate caspase activity independently [47]. In this paper, we confirmed that hHO-1 can directly suppress caspase activity. The anti-inflammatory effect of hHO-1 in hHO-1-Tg fibroblasts was also confirmed by showing suppressed expression of iNOS and RANTES. In the present study we used skin fibroblast isolated from hHO-1-Tg pig for *in vitro* function analysis because our pigs did not express hHO-1 in the endothelial cells. Though most of xenograft rejection processes are initiated from endothelial cell, organs or tissues from our pigs may contribute to improved survival of xenografts. Because the protective effects of hHO-1 are mediated by the products that degradation of heme by hHO-1 [48] and they can be released out from surrounding tissues [49] and affect on endothelial cells. Accordingly, hHO-1 induction in porcine tissues may contribute to improved survival of xenografts.

Although hHO-1 has potent anti-apoptotic and multiple other protective functions, the actions of this one molecule may not be sufficient to block cell death in the transplant setting. This requires further evaluation in transplant models. Several other anti-apoptotic genes that block other lethal pathways including SOD, Bcl-2, BAG-1, catalase, EPO-1, and XIAP are candidate genes for creating transgenic pigs suitable for xenografts [50]. Oropeza et al. generated hA20 transgenic pigs using a CAG promoter and demonstrated that hA20 transgenic PAECs were resistant to the induction of Fas-mediated apoptosis [44].

In conclusion, we generated three hHO-1-Tg pigs and revealed their expression of the transgene by western blotting and immunohistochemistry. Human HO-1 transduced NPCCs and fibroblasts isolated from hHO-1-Tg pig showed anti-oxidative, anti-apoptotic, and anti-inflammatory properties. Though additional *in vivo* study using pig-to-nonhuman primate model is required for further analysis, the present study strongly suggest that the hHO-1 gene could be a promising choice as part of a multi-gene strategy to prevent AVR or to minimize damage after ischemia/reperfusion.
